# Thyrotoxic Encephalopathy With Seronegative Thyroiditis: An Unusual Occurrence

**DOI:** 10.7759/cureus.98129

**Published:** 2025-11-30

**Authors:** Soorya Bavikeri Shivakumara Hegde, Janak Sureshbhai Nayak, Mahesh Sathiavageeswaran

**Affiliations:** 1 Internal Medicine, University Hospitals of North Midlands NHS Trust, Stafford, GBR; 2 Acute Medicine, University Hospitals of North Midlands NHS Trust, Stafford, GBR; 3 Endocrinology, Diabetes and Metabolism, University Hospitals of North Midlands NHS Trust, Stafford, GBR

**Keywords:** first instance, middle-aged females, seronegative thyroiditis, seronegativity, thyrotoxic encephalopathy

## Abstract

Thyrotoxic encephalopathy is a rare phenomenon that is associated with autoimmune thyroid disorders (Hashimoto’s thyroiditis or Graves’ disease), where elevated levels of antithyroid antibodies with an excellent response to steroids are seen. Here, we describe a case of thyrotoxic encephalopathy due to seronegative thyroiditis. There are no reports of encephalopathy occurring in a patient with thyrotoxicosis in the absence of thyroid antibodies in the English literature.

A 50-year-old woman who was admitted with fever and subclinical hyperthyroidism with negative antibodies progressed over a period of 10 days to develop acute confusion and frank hyperthyroidism. Treatment was started with carbimazole; however, following negative antibodies and imaging studies in keeping with thyroiditis, carbimazole was stopped. Her symptoms worsened, and thyroid function tests continue to show hyperthyroidism. She was extensively investigated for encephalopathy by a multidisciplinary team comprising an endocrinologist, a neurologist, and an infectious diseases team, and neurological causes were comprehensively ruled out. She was restarted on carbimazole, and over the next seven days, her confusion settled, and she returned to her baseline functioning. A diagnosis of thyrotoxicosis secondary to seronegative thyroiditis was made. Over the subsequent few weeks, carbimazole was withdrawn, and she was started on levothyroxine. She continued to make good progress.

We present the first case of seronegative thyrotoxicosis and a review of the literature. It is important to consider thyrotoxicosis as a cause for unexplained encephalopathy and to remember that it can be seen with seronegative thyroiditis. It is a diagnosis of exclusion with treatment involving antithyroid drugs.

## Introduction

Thyrotoxic encephalopathy is a rare clinical entity associated with two autoimmune thyroid disorders: Hashimoto’s thyroiditis (more commonly) or Graves’ disease [1-3]. It is more prevalent in women and presents with altered sensorium, behavioral or cognitive disturbances, and, rarely, psychosis or Parkinsonism [4-6]. The mechanisms remain poorly understood, although encephalopathy associated with autoimmune thyroid disease is thought to involve cerebral vasculitis with endothelial inflammation or immune complex deposition, leading to global or focal hypoperfusion in the brain [7,8]. It is considered a diagnosis of exclusion [9].

It is characterized by high levels of free tri-iodothyronine (fT3) and free tetra-iodothyronine (fT4), with suppressed thyroid-stimulating hormone (TSH) and high titers of thyroid autoantibodies such as thyroid-stimulating hormone receptor antibodies (TRAb), thyroglobulin antibodies (Tg), and antithyroid peroxidase antibodies (TPOAb) [10]. In a pooled analysis of several published studies [11], 71 HE patients had TPOAb measured - 67 of them (≈ 94%) were positive. In the same review, the authors estimated the sensitivity of anti-TPO for Hashimoto's encephalopathy to be ~85.7% and the specificity to be 60.6%. Tg positivity was seen in 6/13 (≈46.2%) instances in a case series [12]. There is a significant improvement in symptoms following the administration of corticosteroids [13-15].

## Case presentation

First admission

A 50-year-old woman attended the emergency department with a three-week history of feeling unwell, lassitude, lethargy, loss of appetite, intermittent fever up to 39°C, and non-radiating pain over the anterior part of her neck, brought on by her cough, diffuse in nature, and not associated with neck stiffness or sensitivity to light. She did not report any change in bowel habits (such as constipation). Twenty-four hours before admission, she had two episodes of non-projectile vomiting. Her general practitioner examined her and also performed a naso-endoscopic examination, which was reported to be normal. Her general practitioner advised her to increase the dose of her lansoprazole, but this did not make a difference.

Three months earlier, she had undergone an uncomplicated sleeve gastrectomy. Her medications included lansoprazole, citalopram (for depression), and vitamins. On examination, she had tenderness in the center of her neck. Her vital signs at this time included a heart rate of 68 beats per minute, a respiratory rate of 18 cycles per minute, a blood pressure of 108/73 mm Hg (millimeters of mercury), oxygen saturation of 94% on room air, and a temperature of 36.9°C.

The thyroid was not enlarged, and there was mild left-sided lymphadenopathy. She was noted to be cognitively well. Blood tests were done and are enclosed in Table 1. Significant findings were very mild neutrophilia and suppressed TSH levels with normal free thyroxine.

**Table 1:** Results of initial blood tests *mIU/L stands for milli-international units per liter. **pmol/L stands for picomoles per liter. ***IU/L stands for international units per liter. ****mmol/L stands for millimoles per liter.

Parameter	Values (First admission)	Values (Second admission)	Values (Third admission)	Reference range (units)
Thyroid-stimulating hormone (TSH)	0.04	<0.01	<0.01	0.38-0.53 mIU/L*
Free tri-iodothyronine (fT3)	6.4	15.4	7.0	3.5-6.5 pmol/L**
Free tetra-iodothyronine (fT4)	22.0	63	30.3	11.5-22.7 pmol/L
Thyroid receptor antibody (TRAb)	-	<0.03	<0.03	0.0-0.90 IU/L***
Thyroid peroxidase antibody (TPOAb)	-	Negative	Negative	Negative
Thyroglobulin antibody (TgAb)	-		20	<40
Sodium	140	141	138	133-146 mmol/L****
Potassium	4.1	3.7	3.7	3.5-5.3 mmol/L
Urea	5.2	5.1	2.7	2.5-7.8 mmol/L
Creatinine	61	53	41	45-84 micromoles per liter
Estimated glomerular filtration rate (eGFR)	>90	>90	>90	>90 milliliters per minute per 1.7
Magnesium	0.71	0.72	0.68	0.70-1.00 mmol/L
Inorganic phosphate	0.71	0.61	0.81	0.80-1.50 mmol/L
Adjusted calcium	2.56	2.70	2.69	2.20-2.60 mmol/L
C-reactive protein	118	89	44	<4 milligrams per liter
Albumin	30	30	33	35-50 grams per liter
Alkaline phosphatase	99	86	93	30-130 IU/L
Alanine aminotransferase	18	19	94	0-34 IU/L
Serum bilirubin	11	10	7	0-21 micromoles per liter
Hemoglobin	117	121	134	115-165 grams per liter
White cell count	12.80	10.80	11.20	4-11 10^9^ per liter
Neutrophils	10.11	8.10	8.02	2.0-7.5 10^9^ per liter
Platelets	303	332	336	150-450 10^9^ per liter
Cytomegalovirus serology		Weak reactivity	Weak reactivity	-

The differentials included thyroiditis, gastro-esophageal reflux disease (due to a history of bariatric surgery), and infection of unknown source. Endocrinology review noted that her thyroid function tests two years earlier had been within normal limits, and with the current thyroid picture and suspected thyroiditis, repeat thyroid function testing was planned in six weeks. Empiric broad-spectrum antibiotics were administered for 24 hours. Repeat examination of the neck did not reveal any goiter. An infectious disease consultant review was carried out, and they agreed that the diagnosis was thyroiditis, causing her fever and inflammatory response. She was reassured and discharged.

Second admission

Thirteen days following discharge, she was brought back to the hospital by her husband with complaints that she had been confused for 24 hours. There was no complaint of headache. Her heart rate was 98 beats per minute, with a respiratory rate of 18 and blood pressure of 120/70 mm Hg. Her temperature was 38°C. Examination showed her to be alert but mildly confused. She was shivering. No neck stiffness or rash was elicited. No focal neurological abnormality could be demonstrated. Blood tests showed that the renal and liver profiles were normal, and the C-reactive protein (CRP) was raised (89 mg/L) but was slowly settling down from previous levels. The tests are shown in Table 1. Cytomegalovirus (CMV) IgM was detected as weakly positive. Parvovirus B19 IgG and Epstein-Barr virus (EBV) IgG were detected on previous tests. Computed tomogram (CT) scans of the brain, thorax, abdomen, and pelvis were all reported as normal. A magnetic resonance imaging (MRI) scan of the brain with contrast was also reported as normal. The differentials included viral encephalitis, autoimmune encephalopathy, and metabolic encephalopathy.

**Figure 1:**
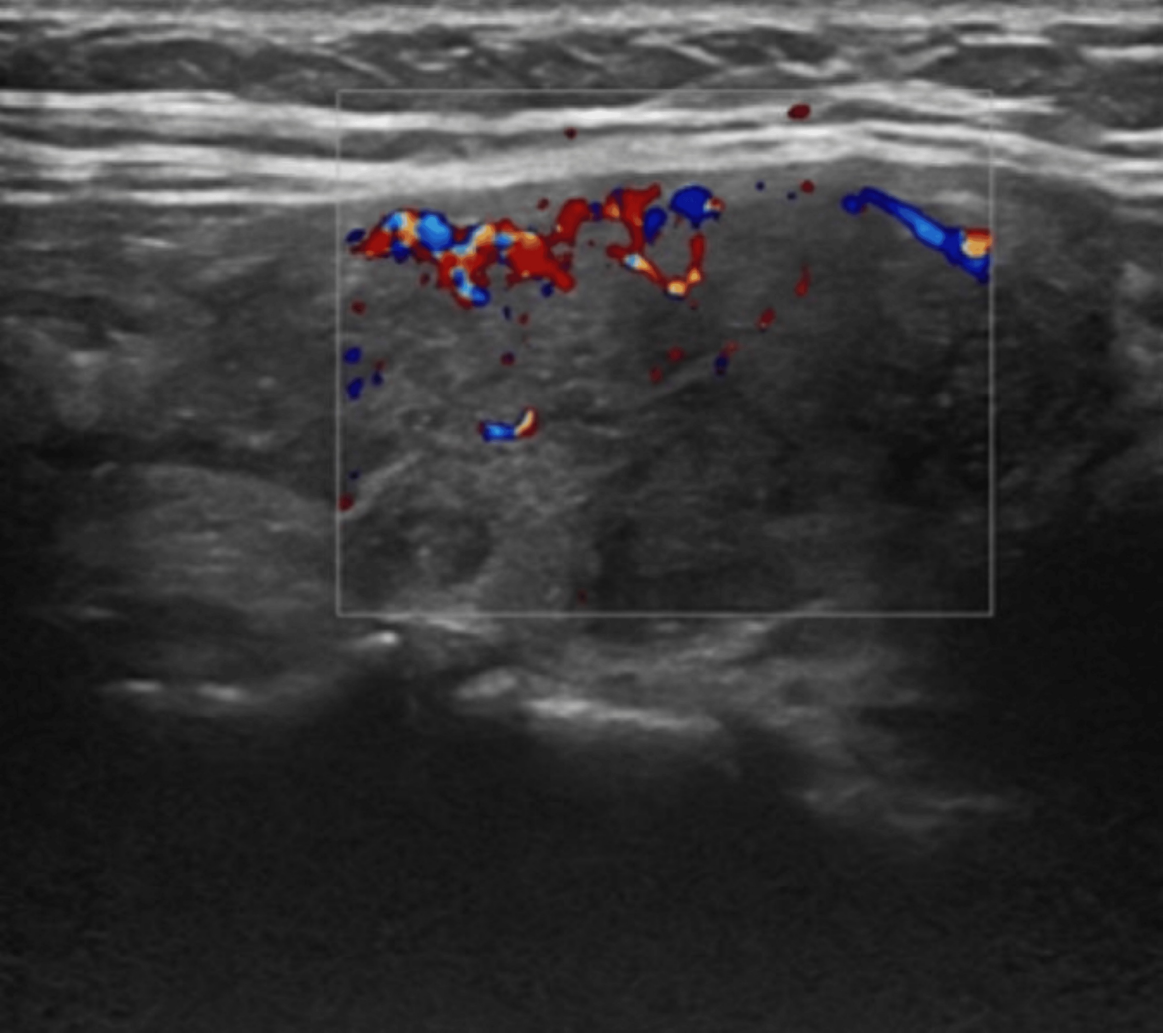
Ultrasound scan of the right lobe of thyroid with overlaid Doppler depicting coarse echotexture and diffuse disease

**Figure 2:**
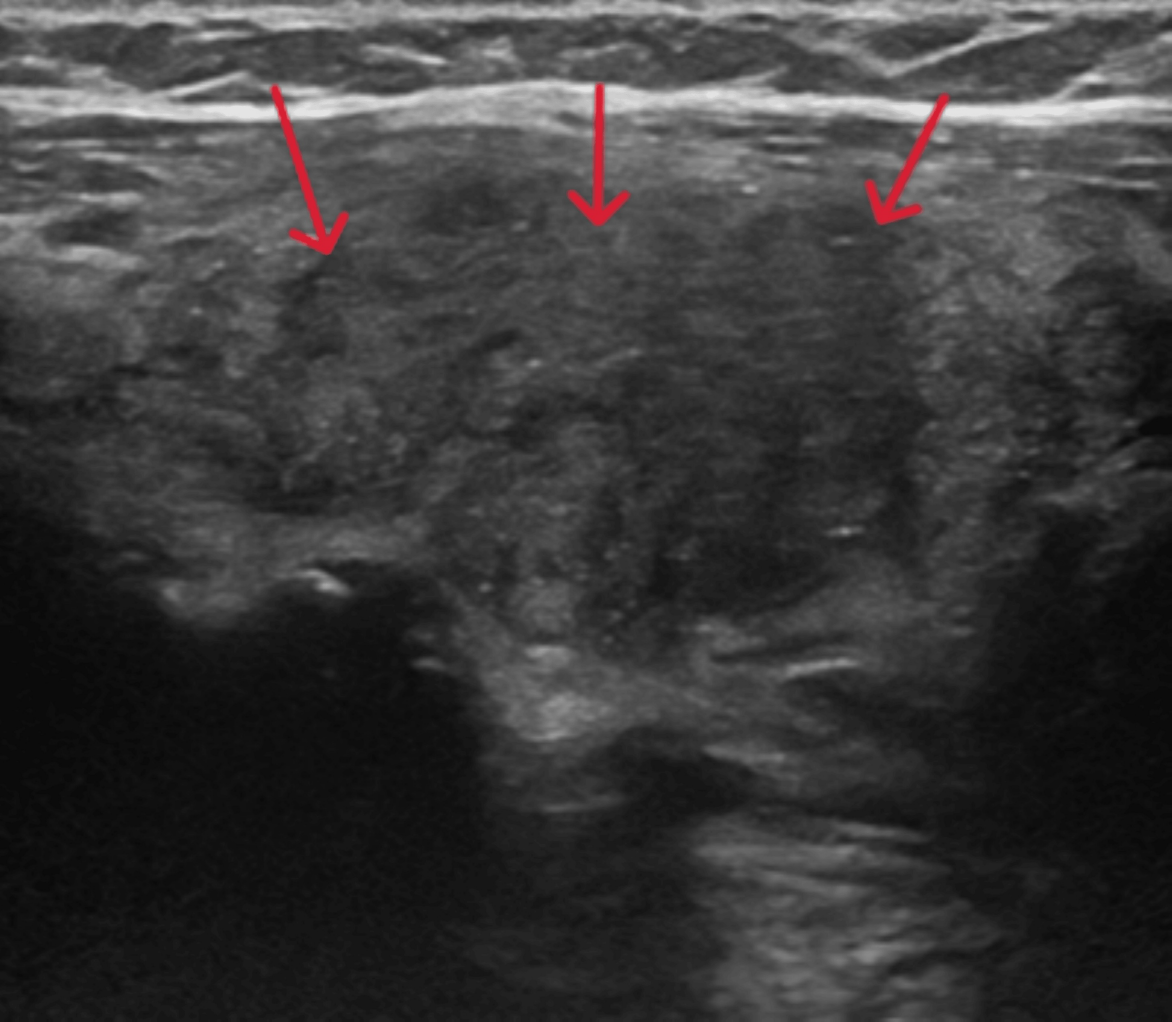
Ultrasound scan of the left lobe of thyroid depicting a bulky thyroid (red arrows) with diffuse disease

Thyroid function tests showed that the TSH was now fully suppressed, with free tetra-iodothyronine (fT4) of 63 pmol/L (11.5-22.7) and free tri-iodothyronine (fT3) of 15.4 pmol/L (3.5-6.5). Thyroid antibody testing was negative, as shown in Table 1. She was therefore started on carbimazole tablets 60 milligrams (mg) once daily. An ultrasound scan of the thyroid (Figures 1, 2) demonstrated a bulky and hypoechoic thyroid with coarse echotexture consistent with diffuse disease.

**Figure 3:**
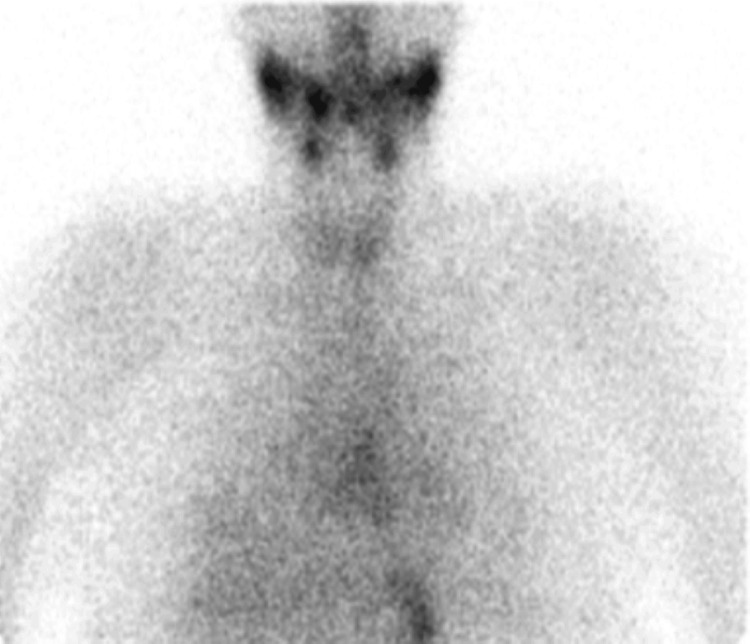
Thyroid nuclear medicine pertechnetate scan (planar view) depicting reduced uptake of radiotracer

**Figure 4:**
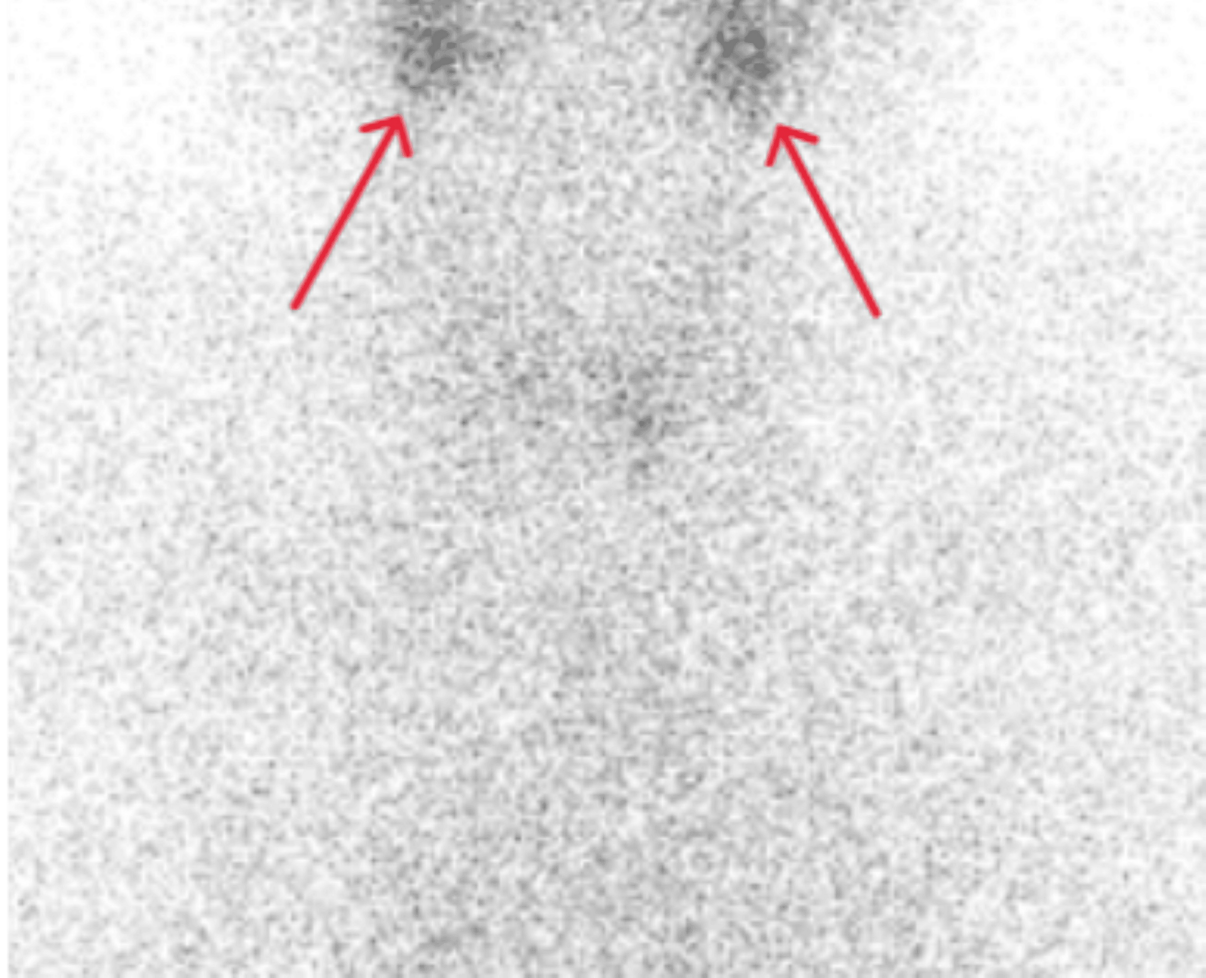
Thyroid nuclear medicine pertechnetate scan (zoomed anterior neck view) depicting reduced uptake of radiotracer (red arrows) with preserved background activity

The submandibular and parotid salivary glands were unremarkable. No cervical lymphadenopathy was reported. Thyroid pertechnetate scan (Figures 3, 4) showed reduced thyroid uptake with preserved background activity consistent with thyroiditis. No features of Graves’ disease were demonstrated. MRI diffusion weighted scan showed unremarkable appearances of the brain. In view of these findings, the carbimazole 20 milligram once-a-day tablet was stopped after being given for approximately two weeks. The patient was reassured and discharged home when her confusion settled.

Third admission

Two days after discharge from the hospital, the confusion recurred. The patient became completely disoriented in time, place, and person, along with behavioral changes where she could not stop laughing and could not comprehend anything her husband was saying. She also had short-term memory loss and could not recognize her husband. She reported that her vision was blurry. There had been no recurrence of fever, but she seemed to complain of pain in her neck. On examination, she had a vacant look and did not answer any questions asked of her. There was no focal neurological deficit, and she had preserved power tone and reflexes in all limbs. No cerebellar signs were detected. The thyroid glands were mildly swollen with mild tenderness. The rest of the examination was normal. Blood tests are shown in Table 1.

A repeat CT scan of the brain did not show any abnormality. Repeat thyroid function tests showed that TSH was suppressed, and the free T4 was now 30.3 pmol/L, compared to 63 pmol/L four days earlier. Repeat testing for thyroid antibodies, including thyroglobulin antibodies (TgAb), was negative. With a clinical picture of acute encephalitis, she was started on intravenous acyclovir at 10 milligrams per kilogram (mg/kg), and carbimazole was restarted at a lower dose of 20 mg daily. The differentials included structural central nervous system (CNS) lesions, infectious encephalopathy, and autoimmune (including Hashimoto’s) encephalopathy.

She was reviewed by a neurologist who felt that she had some disorientation in terms of time, with no other demonstrable focal neurological deficits. Acyclovir was stopped. Further tests looking for other causes of encephalopathy were carried out, including liver function tests, repeat thyroid antibody tests, MRI scan, CSF (cerebrospinal fluid) testing, autoimmune encephalitis/antibody screen, heavy metal exposure testing, vasculitis screen, and Lyme disease serology, the results of which are shown in Table 2. A lumbar puncture was performed for CSF testing, and the results are shown in Table 3.

**Table 2:** Results of further blood tests

Parameter	Values	Reference range (units)
Erythrocyte sedimentation rate (ESR)	13	2-16 millimeters per hour
24-hour urine normetadrenaline	0.31	0.00-2.29 micromoles per 24 hours
Urine metadrenaline	0.18	Micromoles per liter
24-hour urine metadrenaline	0.13	0.00-1.49 micromoles per 24 hours
Cortisol	463	Nanomoles per liter
Anti-extractable nuclear antigen antibodies (include Ro, La, Smith, RNP, Jo1, and Scl70)	Negative	-
Ebstein-Barr virus serology	IgG positive	-
Parvovirus B19 serology	IgG positive	-
Cytomegalovirus polymerase chain reaction (PCR)	Not detected	<500 international units per millimeter
Antinuclear antibodies	Negative	-
Antimyeloperoxidase antibodies	0.7	<3.5 units
Antiproteinase 3 antibodies	<0.3	<2 units
Human immunodeficiency virus (HIV) 1 and 2 - antigens and antibodies	Not detected	-
Cardiolipin immunoglobulin G antibody	3.5	<10 units per milliliter
Borrelia burgdorferi IgG/IgM (enzyme immunoassay)	Negative	-
Procalcitonin	<0.03	<0.05 micrograms per liter
Selenium	0.62	0.9-1.7 micromoles per liter

**Table 3:** Results of cerebrospinal fluid testing

Parameter	Values	Reference range (units)
Lactate	1.6	1.1-2.4 millimoles per liter
Glucose	3.2	2.5-4.5 millimoles per liter
Total protein	0.27	0.15-0.45 grams per liter
Red blood cells	79	-
White blood cells	2	0-5 10^9^/L
Organisms	No organisms seen	-
Culture	No growth	-
Anti-N-methyl-D-aspartate receptor	Negative	-
Alpha-amino-3-hydroxy-5-methyl-4-isoxazolepropionic acid receptor 1 and 2 antibody	Negative	-
Dipeptidyl-peptidase–like protein-6 antibodies	Negative	-
Contactin-associated protein 2	Negative	-
Leucine-rich glioma inactivated protein 1	Negative	-
Gamma-aminobutyric acid receptor type B1	Negative	-

Following the negative results from the lumbar puncture and antibody testing, the neurologist was happy to rule out autoimmune causes of encephalopathy, and their clinical impression was that the patient had thyrotoxic encephalopathy. Antithyroid antibodies were repeated, which were once again negative. The electroencephalogram (EEG) showed frequent episodes and runs of small delta waves. These were not associated with clinical manifestations. The neurologist felt that this picture was reflective of underlying thyrotoxic encephalopathy. Opinion was also sought from the infectious diseases consultant, who felt that the previous CMV serology was not clinically relevant. The two serological samples were similar with no change, suggesting cross-reactivity. PCR for CMV was negative, which was also in agreement with this conclusion.

As the CSF was normal, there was no CNS infection causing the confusion. The infectious diseases consultant felt that thyroid inflammation and the contributory rise in thyroid hormones likely led to the confusion. On day two of her admission, she still had visual hallucinations where she saw random people, images, and words that looked twisted or tented and jumping around. She also reported something occasionally blocking her vision. She remained confused. After 72 hours and after three doses of carbimazole (40 mg each given once daily), she was alert and oriented. Her neurological and cognitive improvement continued. Ten days after admission, she was discharged with the final diagnosis of resolving thyrotoxic encephalopathy.

Outcome and follow-up

Two weeks following discharge, her thyroid function tests were repeated, and she was seen in the outpatient clinic. She was able to perform activities of daily living (bathing, eating, and dressing) independently. While her general well-being had improved, she continued to experience interrupted sleep. She complained of hair loss and constipation. She was on a progesterone implant and did not have any periods. She did not feel that her skin was hardened.

Thyroid function testing now showed a TSH of 32.14 mIU/L (0.38-5.33), free thyroxine of 7.1 pmol/L (11.5-22.7), and tri-iodothyronine of 2.9 pmol/L (3.5-6.5), and carbimazole was stopped. A further thyroid function test was done three weeks later, when her TSH was 5.57 mIU/L, and free T4 was 12 pmol/L. She was clinically well. Levothyroxine 25 micrograms once a day was commenced. Further review has been arranged.

## Discussion

Steroid-responsive encephalopathy associated with autoimmune thyroiditis, also known as Hashimoto’s encephalopathy, is the most well-documented form of thyroid-related encephalopathy. Diagnosis of thyrotoxic encephalopathy remains challenging due to its severity and nonspecific presentation. Acute or subacute onset of cognitive impairment, psychiatric symptoms, or seizures with evidence of thyrotoxicosis and neuroimaging that shows normal or nonspecific features are helpful. EEG is often abnormal, showing diffuse lowing or epileptiform activity. It is important to exclude other causes of encephalopathy [8]. A systematic review of 251 cases revealed key findings [16] as noted in Table 4.

**Table 4:** Key clinical features from a systematic review Source: Laurent et al. [16].

Symptom	Frequency
Seizures	47%
Confusion	46%
Memory impairment	43%
Speech disorders	37%

Liu et al. [17] noted in their paper on nine cohort studies involving 49,218 participants that subclinical hypothyroidism was associated with an increased risk of dementia, suggesting that even mild thyroid hypofunction may have detrimental effects on cognitive function, potentially contributing to encephalopathic states. Several mechanisms may contribute to encephalopathy in the context of thyrotoxicosis. Autoimmune process in the presence of antithyroid antibodies, particularly antithyroid peroxidase antibody, may play a role in neurological dysfunction [18]. Thyrotoxicosis can alter cerebral blood flow, potentially leading to neurological symptoms [16]. Excess thyroid hormones may also directly affect brain function and metabolism [19].

The management approach includes addressing the underlying thyroid dysfunction. Immunosuppression with corticosteroids has been shown to have efficacy in steroid-responsive encephalopathy associated with thyrotoxicosis and may be beneficial in thyrotoxic encephalopathy with an autoimmune component [16]. Supportive care includes management of symptoms such as seizures or psychiatric manifestations [20].

We have presented a case of a 50-year-old woman presenting with a prodrome of high temperature, neck pain, tiredness, and lassitude. Ten days later, she developed confusion associated with frank thyrotoxicosis, with free thyroxine levels around three times the upper limit of normal and free T3 about 2.5 times the upper limit of normal, associated with suppressed TSH, consistent with biochemical thyrotoxicosis. She did not have any tremor, tachycardia, diarrhea, sweats, or eye signs. Instead, she was confused and exhibited behavioral changes. There was some initial improvement with antithyroid medications, but as her clinical picture was in keeping with seronegative thyroiditis, perhaps viral thyroiditis, the antithyroid medication was stopped. Seventy-two hours later, there was a recurrence of symptoms, now including behavioral changes, alternating laughing and crying, inability to identify and recognize near and dear ones, and significant memory loss. There was improvement in her cognition and psychiatric symptoms after restarting antithyroid medications.

We meticulously went through the process of ruling out all primary and secondary brain-related causes. Serological and imaging studies were carried out to rule out any other cause of encephalopathy. CT brain and MRI brain did not show any specific features, while the EEG showed nonspecific slow-wave features consistent with thyrotoxic encephalopathy. The antithyroid medications were restarted, and, as suspected, her thyroid function was controlled. She eventually became hypothyroid, after which she was started on levothyroxine, which was associated with clinical improvement. This is the first case report of seronegative thyroiditis associated with thyrotoxic encephalopathy in the English-language literature.

In our case, after ruling out all other possible causes, we were left with only a thyroid abnormality as the cause of her presentation.

## Conclusions

Thyrotoxic encephalopathy is a rare occurrence secondary to Hashimoto’s thyroiditis and Graves’ disease. Based on this case of seronegative thyrotoxicosis that caused profound thyrotoxic encephalopathy and settled following treatment of thyrotoxicosis, non-autoimmune causes of thyroiditis should also be considered when investigating encephalopathy of unknown cause. It is important to rule out other causes of encephalopathy and conduct relevant investigations at the first instance to avoid delays in diagnosis or the institution of appropriate treatment. Treatment involves optimization of thyroid activity with antithyroid medications, and it is vital to be aware of relapses.
